# Retraction: ATG4A promotes tumor metastasis by inducing the epithelial-mesenchymal transition and stem-like properties in gastric cells

**DOI:** 10.18632/oncotarget.28795

**Published:** 2025-12-31

**Authors:** Shi-Wei Yang, Yi-Fang Ping, Yu-Xing Jiang, Xiao Luo, Xia Zhang, Xiu-Wu Bian, Pei-Wu Yu

**Affiliations:** ^1^Department of General Surgery and Center of Minimal Invasive Gastrointestinal Surgery, Southwest Hospital, Third Military Medical University, Chongqing, China; ^2^Key Laboratory of Tumor Immunopathology of Ministry of Education of China, Third Military Medical University, Chongqing, China; ^3^Institute of Pathology and Southwest Cancer Center, Southwest Hospital, Third Military Medical University, Chongqing, China

**This article has been retracted:** Oncotarget has completed its investigation of this article. The investigation shows duplication in Figures 1, 2 and 5. Corresponding author Peiwu-Yu supplied files for a correction. However, it was then discovered that the gastric cancer cell lines SGC-7901 and MGC-803, used in this study, have been reported as contaminated [1–4]. Given the extent of the identified issues, the editors have lost confidence in the data presented and the article’s conclusions can no longer be considered reliable. Therefore, the Editorial decision was made to retract this paper. The authors have been informed of the concerns and the decision to retract. The authors have not acknowledged that their data has been compromised and their conclusions are invalid.

Original article: Oncotarget. 2016; 7:39279–39292. 39279-39292. https://doi.org/10.18632/oncotarget.9827
